# A Case Report of an Adverse Outcome: Development of a Dural Arteriovenous Fistula Following Foramen Magnum Decompression for Chiari Malformation

**DOI:** 10.7759/cureus.83509

**Published:** 2025-05-05

**Authors:** Eitaro Okumura, Motoo Kubota, Ryo Hashimoto

**Affiliations:** 1 Spinal Surgery, Kameda Medical Center, Chiba, JPN

**Keywords:** chiari 1 malformation, dural arteriovenous fistula (davf), foramen magnum decompression, occipital sinus, poor outcome

## Abstract

Chiari malformation is a congenital condition defined by anatomical abnormalities at the craniovertebral junction with downward displacement of cerebellar structures. While some cases are asymptomatic, others present with diverse clinical manifestations, including cerebellar dysfunction, brainstem compression, headaches, hydrocephalus, myelopathy, oropharyngeal dysfunction, sleep-related breathing disorders, scoliosis, and syringomyelia. Surgical intervention is the only treatment option for symptomatic patients, involving foramen magnum decompression (FMD) to decompress the craniovertebral junction and normalize cerebrospinal fluid circulation. Cervical laminectomy and duraplasty may be added depending on the severity of cerebellar tonsil herniation and brainstem compression. Complications of duraplasty include pseudomeningocele formation, cerebrospinal fluid leakage, acute postoperative hydrocephalus, and meningitis. We report a devastating case of a pediatric patient with Chiari malformation who developed cerebellar hemorrhage from a dural arteriovenous fistula (dAVF) after FMD, resulting in death. A 16-year-old female was diagnosed with scoliosis, Chiari malformation, and syringomyelia at age 4. At age 15, due to the progression of scoliosis, corrective fusion surgery was planned, but surgical treatment for Chiari malformation was performed first. The surgery involved FMD, during which a well-developed occipital sinus was observed. When performing duraplasty in addition to FMD and upper cervical laminectomy, the occipital sinus was ligated to reduce bleeding risk. The postoperative course was uneventful with no obvious neurological deficits, and the patient was discharged home. The corrective fusion surgery for scoliosis was also successfully completed, and outpatient follow-up continued. One year and five months after the FMD surgery, the patient presented with a sudden headache and vomiting. Brain MRI revealed cerebellar hemorrhage and suspected dAVF to the occipital sinus. Cerebral angiography showed the occipital artery as the main feeder, the paramedian posterior marginal sinus as the shunt point, and the occipital sinus as the main drainer. Due to deep venous reflux indicating high risk of rebleeding, transarterial and transvenous embolization were performed. However, cerebral edema progressed, hydrocephalus worsened, and cerebellar hemorrhage recurred, resulting in the patient's death. When performing FMD in pediatric cases, thorough preoperative evaluation of venous sinuses is essential. Care should be taken to avoid ligation when a well-developed occipital sinus is observed. In such cases, consider limiting the procedure to bony decompression only.

## Introduction

Chiari malformation is a congenital developmental disorder of the nervous system. Chiari malformation type I is one of the most common clinical subtypes, characterized by cerebellar tonsil herniation of more than 5 mm below the foramen magnum plane due to abnormalities in bone and brain tissue structures, sometimes accompanied by syringomyelia and neurological abnormalities [1]. Syringomyelia occurs in 60%-85% of patients with Chiari malformation type I, which can cause various symptoms including cerebellar dysfunction, brainstem compression, headaches, hydrocephalus, myelopathy, oropharyngeal dysfunction, sleep-related breathing disorders, and scoliosis [2,3]. Many of these symptoms tend to worsen over time [2,3]. Surgical intervention is the only recommended treatment for symptomatic Chiari malformation type I, with various surgical methods currently available, including simple foramen magnum decompression (FMD), FMD with duraplasty, and FMD combined with the resection of tonsils [4]. In particular, surgery involving duraplasty may be associated with complications such as pseudomeningocele formation, cerebrospinal fluid leakage, acute postoperative hydrocephalus, and meningitis. To date, there have been no previous reports of dural arteriovenous fistula (dAVF) as a postoperative complication of Chiari malformation surgery. We report a devastating case of a pediatric patient with Chiari malformation type I who developed cerebellar hemorrhage from a dAVF after FMD with duraplasty, resulting in death.

## Case presentation

**Figure 1:**
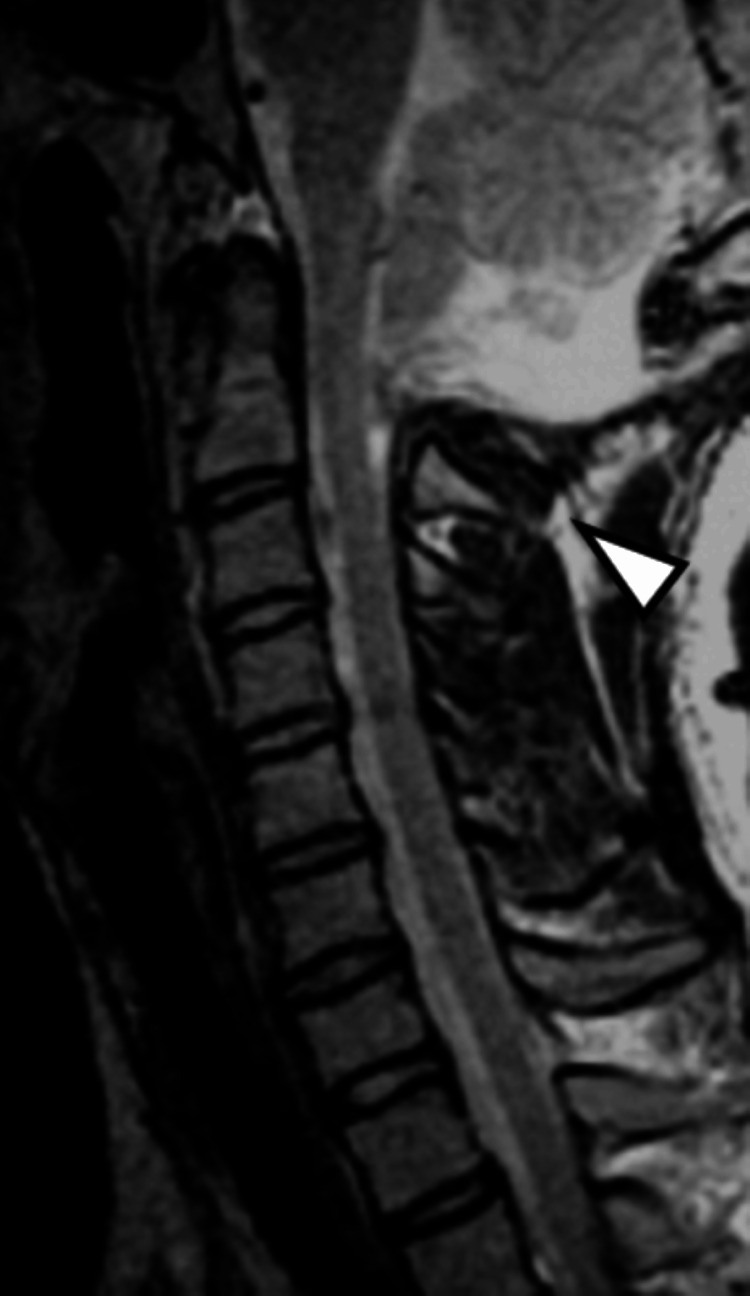
Post-foramen magnum decompression MRI (T2-weighted sagittal view). Foramen magnum decompression, C1 laminectomy, and duraplasty were performed for Chiari malformation type I. Cerebellar tonsillar herniation has improved (arrowhead).

A 16-year-old female was diagnosed with Chiari malformation type I, syringomyelia, scoliosis, and sleep apnea syndrome at age 4. Brace therapy for scoliosis was initiated at age 9, but the scoliosis continued to worsen. Therefore, at age 15, corrective fusion surgery was planned. Before this, surgical treatment for Chiari malformation was performed. FMD with duraplasty was planned. During the operation, after performing FMD and C1 laminectomy, a well-developed occipital sinus was observed. Considering the risk of bleeding during duraplasty, the occipital sinus was ligated. The postoperative course was uneventful with no obvious neurological deficits, and the patient was discharged home. As the cerebellar tonsil herniation improved (Figure 1), corrective fusion surgery for scoliosis was performed three months after the FMD.

**Figure 2:**
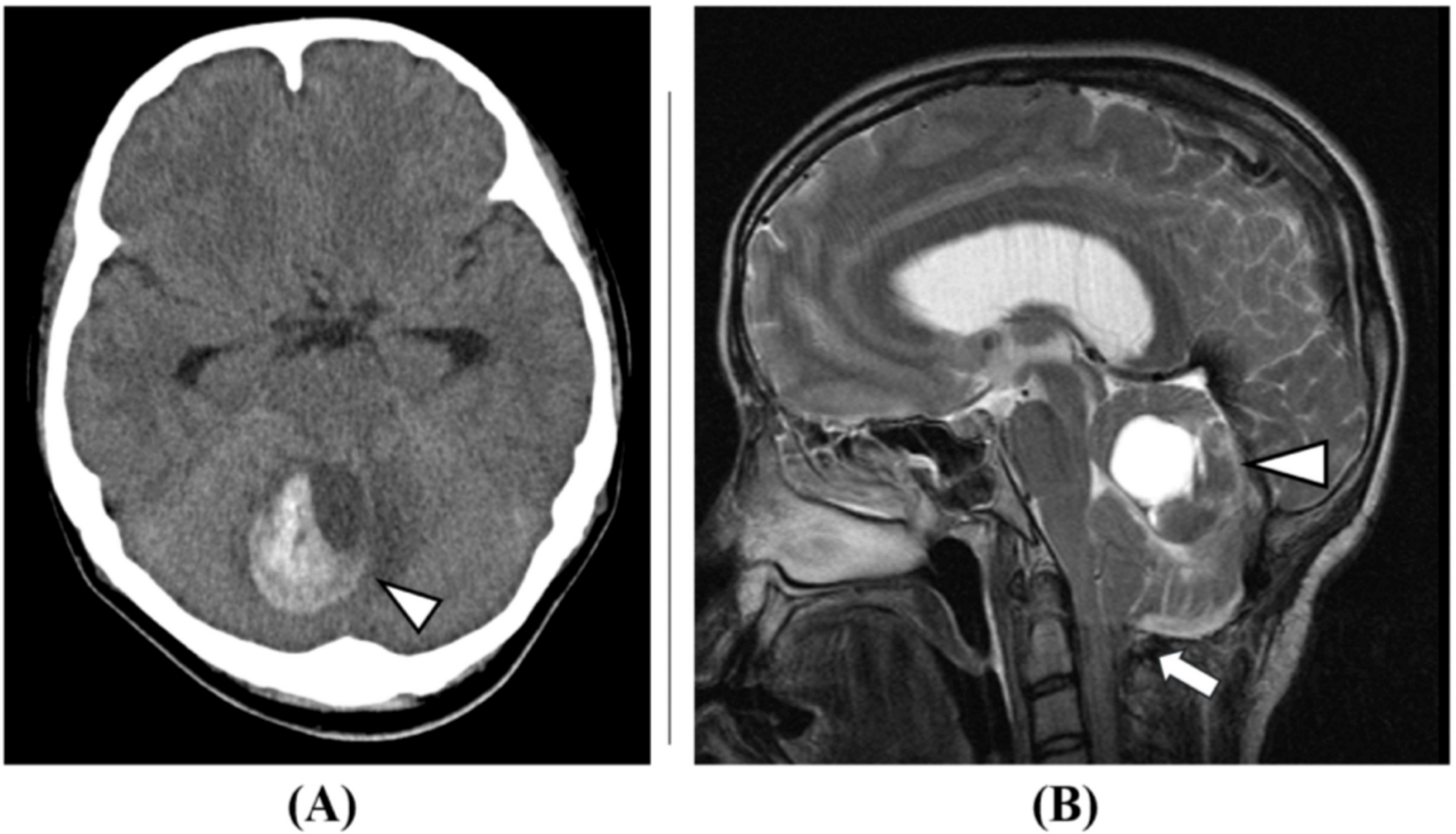
Brain imaging studies (one year and five months after foramen magnum decompression): (A) brain CT image (axial view) and (B) brain MRI image (T2 sagittal). A cerebellar hemorrhage with a maximum diameter of 42 mm was observed (arrowheads in A and B). The cerebellar tonsil herniation has improved (arrow in B).

The patient continued outpatient follow-up. Brain MRI one year and one month after FMD showed a small cerebellar hemorrhage; however, as the patient was asymptomatic, observation was continued. One year and five months after the FMD, the patient was admitted emergently with headache and nausea. Imaging studies revealed an enlargement of the cerebellar hemorrhage (Figure 2), and a dAVF to the occipital sinus was suspected.

**Figure 3:**
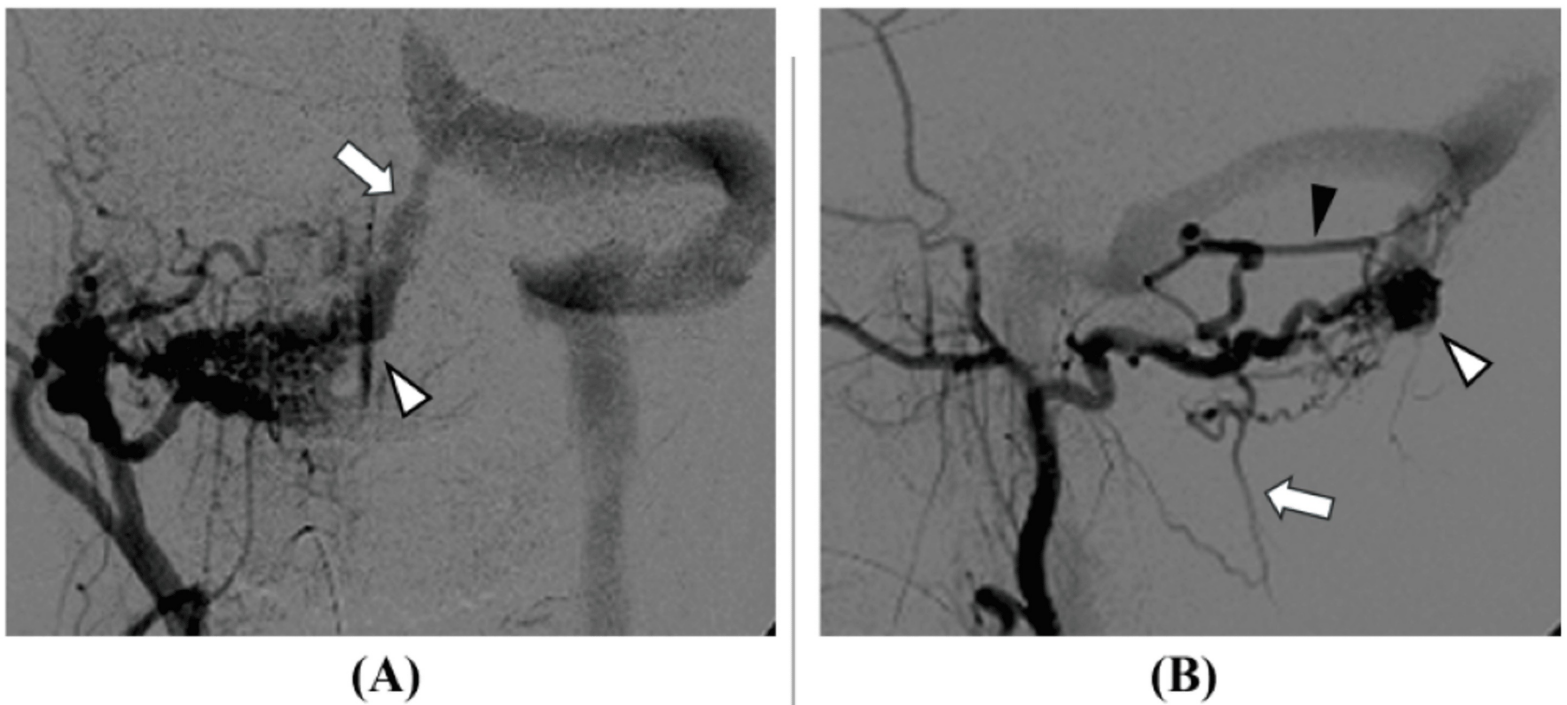
Cerebral angiography images (before treatment): (A) frontal view and (B) lateral view. The occipital artery (arrowhead in A) was the main feeder, with the paramedian posterior marginal sinus (arrow in A, and white arrowhead in B) as the shunt point, and the occipital sinus as the main drainer. Deep venous reflux into the deep venous system, including the median posterior medullary vein (arrow in B) and inferior vermian vein (black arrowhead in B), was present, indicating a high risk of rebleeding. The diagnosis was Cognard type IIa+b dural arteriovenous fistula.

Cerebral angiography showed the mastoid branch of the occipital artery as the main feeder, the paramedian posterior marginal sinus as the shunt point, and the occipital sinus as the main drainer. Deep venous reflux to the deep venous system was present, classified as Cognard type IIa+b, and was determined to be at high risk for rebleeding (Figure 3).

**Figure 4:**
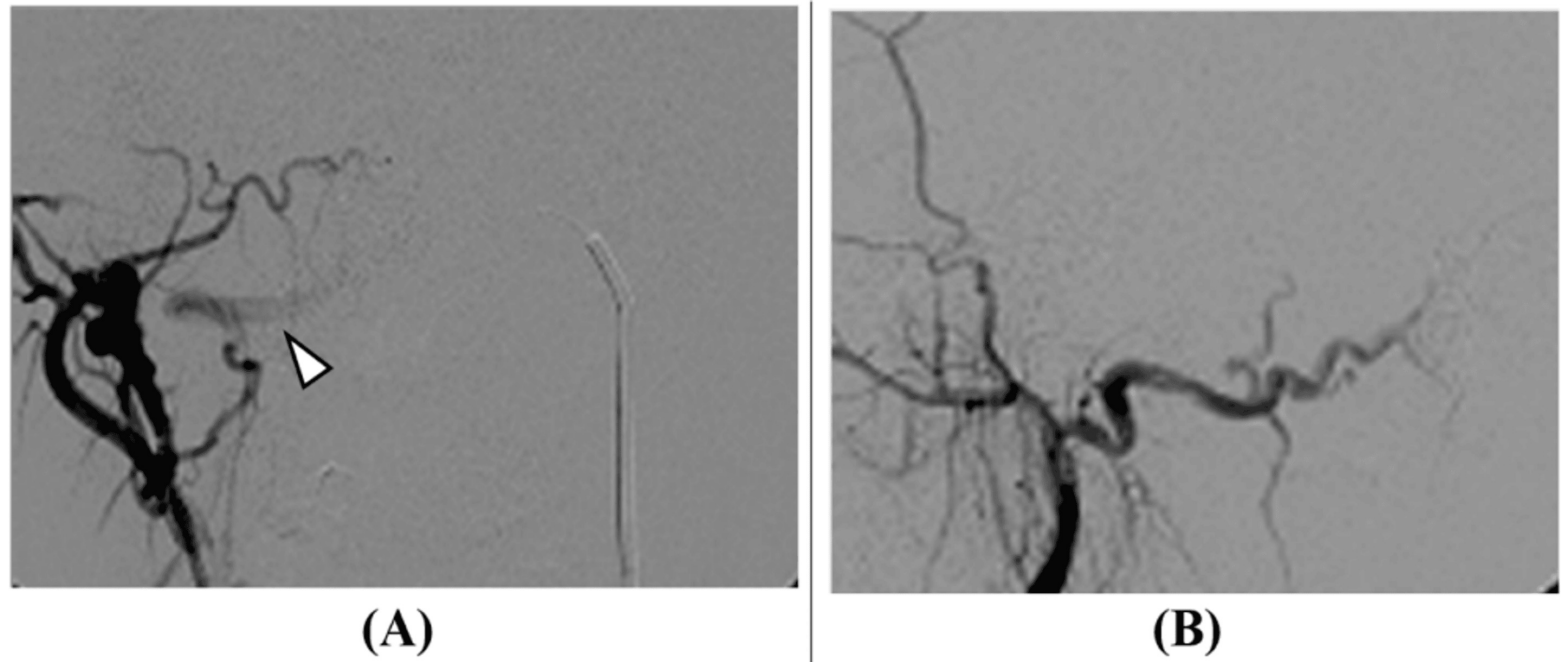
Cerebral angiography images (after treatment). Transarterial embolization from the occipital artery and transvenous embolization from the occipital sinus were performed. The dural arteriovenous fistula largely regressed, though blood flow to the occipital sinus slightly persisted (arrow in A).

Transarterial embolization of the main feeder and transvenous embolization of the shunt point were performed (Figure 4).

**Figure 5:**
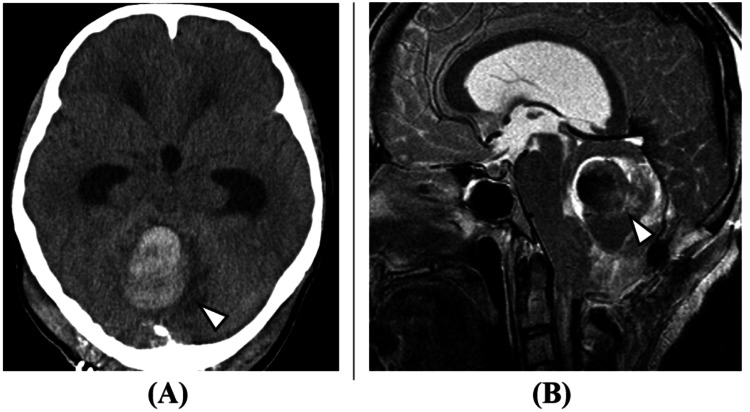
Brain imaging: (A) brain CT image (axial view) and (B) brain MRI image (T2 sagittal). Brain imaging studies conducted four days after neuroendovascular treatment show an increase in the size of the cerebellar hemorrhage, suggesting rebleeding (arrowheads in A and B).

After neuroendovascular treatment, no obvious neurological deficits were noted, aside from occipital headache. However, as blood flow to the occipital sinus slightly persisted, recurrence of cerebellar hemorrhage was observed four days after the neuroendovascular treatment (Figure 5). Craniotomy for hematoma removal and decompressive craniectomy were performed, but the patient died 14 days after the neuroendovascular treatment.

## Discussion

Outcomes for Chiari malformation surgery vary, but many reports indicate improvement or stabilization after FMD with duraplasty. Aghakhani et al. reported on the clinical course of 157 patients with Chiari-related syringomyelia who underwent FMD with duraplasty, noting clinical improvement in 63%, stabilization in 31%, and worsening or death in 6% and 1%, respectively, during a median follow-up period of 88 months [5]. Hoffman et al. reported a perioperative complication rate of 3% for patients with Chiari malformation type I who underwent FMD with duraplasty [6]. Meanwhile, dAVF has been recognized as an acquired condition since Houser et al. and Chaudhary et al. reported its occurrence after venous sinus occlusion [7,8]. There are also reports of new dAVF development after sinus thrombosis in children [9]. Factors thought to contribute to dAVF formation include trauma, hormonal imbalance, venous sinus thrombosis, coagulation abnormalities, and venous hypertension. The possibility of new dAVF appearance due to venous hypertension load has been reported in both animal models and humans [10,11]. In this case, detailed evaluation of venous sinuses was not performed preoperatively, and the possibility of postoperative dAVF development was not considered. Ligation of a well-developed venous sinus may have led to increased venous pressure, resulting in dAVF development. While preserving a well-developed venous sinus during duraplasty would be ideal, if there are concerns about injury and bleeding risk during surgery, one option is to forgo the duraplasty. Yahanda and Limbrick reported that FMD without duraplasty leads to shorter operating times, reduced complications, and improvement in symptoms and syrinx size [12], while Durham and Fjeld-Olenec found that FMD with duraplasty has a lower risk of reoperation compared to FMD alone, although there are no differences in clinical improvement or reduction of syringomyelia [13]. On the other hand, some reports indicate that duraplasty results in lower recurrence rates and greater clinical improvement for Chiari-related syringomyelia [14], suggesting that the surgical approach should be considered on a case-by-case basis. If syringomyelia does not improve with bony decompression alone, additional shunt surgery may be considered. Syringo-subarachnoid (SS) shunt for syringomyelia, although not a fundamental surgical approach to the pathology, has been used in cases where posterior decompression alone does not reduce the syrinx [15,16]. In cases with clearly developed venous sinuses, it is advisable to limit the procedure to bony decompression without ligating the venous sinus. If the syrinx does not reduce, consideration should be given to adding an SS shunt or exploring other approaches.

## Conclusions

We experienced a devastating case of a pediatric patient with Chiari malformation who developed cerebellar hemorrhage from a dAVF after FMD, resulting in death. When performing FMD in pediatric cases, thorough preoperative evaluation of venous sinuses is essential. When a well-developed occipital sinus is observed, ligation should be avoided whenever possible to prevent serious complications.
